# Analysis of genetic polymorphisms in sudden sensorineural hearing loss and artificial intelligence-supported individualized precision therapy

**DOI:** 10.3389/fneur.2025.1643435

**Published:** 2026-02-17

**Authors:** Xin Li, Dong Yang

**Affiliations:** 1 Department of Otolaryngology-Head and Neck Surgery, Beijing Tsinghua Changgung Hospital, School of Clinical Medicine, Tsinghua Medicine, Tsinghua University, Beijing, China; 2 Department of Otorhinolaryngology (ENT), Tianjin Third Central Hospital/Central Hospital, Tianjin University, Tianjin, China

**Keywords:** SSNHL, treatment strategies, genetic polymorphisms, AI models, individualized treatment

## Abstract

**Background:**

Sudden sensorineural hearing loss (SSNHL) is characterized by acute onset, complex pathogenesis, and visible variability in prognosis, making precise treatment challenging. This article focuses on identifying key factors influencing the treatment outcomes of SSNHL.

**Methods:**

Clinical data were collected from 200 SSNHL patients, recording treatment regimens including systemic and intratympanic steroid administration. Genetic polymorphisms were analyzed via blood testing, and a personalized treatment prediction model was constructed and validated. An independent external validation set of 100 cases was included to assess model generalizability. Comparative efficacy predictions were performed among multifactorial logistic regression, convolutional neural network (CNN), random forest, and support vector machine models.

**Results:**

As against systemic steroid therapy, intratympanic injection, and combination treatment (corticosteroids combined with retroauricular subtympanic membrane and intratympanic injections) showed superior recovery rates. The distinction between combination treatment and monotherapy was visible (*p <* 0.01). At the level of key genetic polymorphisms, specific single-nucleotide polymorphism sites in genes such as GJB2, SLC26A4, TNF-*α*, and CYP3A4 were closely associated with treatment responses, with different genetic profiles corresponding to distinct treatment recommendations. In AI-based treatment efficacy prediction, the CNN model demonstrated significantly higher sensitivity, specificity, and accuracy compared to random forest, support vector machine, and other models (*p* < 0.05). It consistently outperformed traditional multifactorial logistic regression in both internal and external validation sets, particularly in identifying poor-recovery cases (*p* < 0.05).

**Conclusion:**

In SSNHL treatment, the combined approach of postauricular subperiosteal and intratympanic steroid injections was significantly more effective than systemic steroid therapy, representing the optimal treatment choice. Specific genetic polymorphisms were closely associated with treatment response and may serve as molecular biomarkers for personalized therapy. The deep learning CNN model exhibited superior performance in efficacy prediction, surpassing conventional models, and could assist in precision treatment decision-making.

## 1 Background

Sudden sensorineural hearing loss (SSNHL), a common otolaryngological emergency with increasing incidence in recent years, poses a significant threat to hearing health and adversely affects patients’ daily life, career development, and mental well-being (1, 2). Characterized by abrupt onset, patients typically experience rapid hearing deterioration in one or both ears within 72 h, often accompanied by tinnitus, aural fullness, and in some cases, vertigo, nausea, and vomiting. The multifactorial pathogenesis involves complex interactions among cochlear microcirculatory disorders, viral infections, immune abnormalities, and inner ear sensory cell damage, creating challenges for precise prevention and treatment (3, 4).

Currently, the clinical management of SSNHL primarily involves corticosteroid-based therapy combined with comprehensive treatment strategies aimed at improving cochlear microcirculation and providing neurotrophic support, with the goal of multidimensional intervention to preserve hearing function (5–7). However, significant interindividual variability in treatment response persists even with standardized protocols: while some patients achieve rapid hearing recovery and resume normal life, others show minimal improvement despite multiple drug combinations and therapeutic approaches, ultimately suffering from persistent hearing impairment and consequent quality-of-life deterioration (8). This pronounced heterogeneity in treatment outcomes has prompted in-depth investigations into underlying mechanisms. The rapid advancement of artificial intelligence (AI) technologies now provides powerful analytical capabilities to address this challenge (4). Through sophisticated deep learning algorithms, AI systems can efficiently process complex multimodal clinical data—integrating genomic profiles, clinical manifestations, and longitudinal monitoring parameters—to identify latent association patterns. Such approaches facilitate the detection of SSNHL-associated genetic polymorphisms and enable personalized outcome prediction by simulating therapeutic responses based on individual patient characteristics, thereby supporting clinicians in making precision treatment decisions (9, 10).

This study integrated cutting-edge genetic analysis technologies with AI algorithms to systematically investigate potential associations between genetic polymorphisms and therapeutic responses, thereby establishing a bridge toward precision medicine. By extensively collecting patient genomic samples and employing AI to construct highly reliable treatment prediction models, this study ultimately aimed to develop personalized optimal treatment strategies for individual SSNHL patients, thereby overcoming current therapeutic limitations.

## 2 Materials and methods

### 2.1 Collection and processing of clinical data

(1) Patient inclusion: 200 patients diagnosed with SSNHL were collected from January 1, 2024, to December 31, 2024. The diagnosis of sudden deafness was based on the criteria established by Chandrasekhar et al. (11) in the *Clinical Practice Guideline: Sudden Hearing Loss (Update)* published in *Otolaryngol Head Neck Surg*. SSNHL was defined as an unexplained, rapidly occurring hearing loss (HL) or reduction, with no other cranial nerve dysfunction beyond the auditory nerve. Patients were screened using the diagnostic criteria established by the Otorhinolaryngology Head and Neck Surgery Branch of the Chinese Medical Association. (2) Hearing assessment: Auditory evaluation of SSNHL subjects was conducted in a sound-proof room meeting acoustic standards, using the Japanese-made AA-79S audiometer. The average hearing level was calculated based on the mean hearing threshold at four frequencies: 500 Hz, 1,000 Hz, 2,000 Hz, and 4,000 Hz. The hearing test results of SSNHL patients were classified according to the standards set by the World Health Organization in 1997. The specific classifications are as follows: (1) Low-frequency decline (LFD) group: Patients with HL at frequencies below 1,000 Hz, or HL of at least 20 dB at both 250 Hz and 500 Hz; (2) High-frequency decline (HFD) group: HL at frequencies above 2,000 Hz, or HL of at least 20 dB at either 4,000 Hz or 8,000 Hz; (3) Full-frequency decline (FD) group: Patients with HL across the entire frequency range from 250 Hz to 8,000 Hz, with an average hearing threshold not exceeding 80 dB; (4) Total deafness (TD) group: An average hearing threshold of 81 dB or higher across the frequency range from 250 Hz to 8,000 Hz. (5) Profound hearing loss: defined as an average hearing threshold no less than 91 dB (across frequencies from 250 Hz to 8,000 Hz). The distinction from the TD group lies in the presence of residual hearing at certain high or low frequencies in profound hearing loss cases (where the TD group is defined by an average threshold of 81–90 dB, and no less than 91 dB signifies profound hearing loss).

### 2.2 Treatment strategies

The treatment regimens administered to patients were documented in detail, including systemic and intratympanic corticosteroid therapy (e.g., methylprednisolone sodium succinate, with an initial systemic dose of 1 mg/kg/day for 7–10 days followed by tapering; intratympanic dexamethasone injection, 5 mg per administration, twice weekly). Vasodilation/hemodilution therapy employed regionally utilized experimental agents in East Asia: *Ginkgo biloba* extract injection (20 mL daily IV infusion, some clinical studies suggest potential antioxidant and microcirculation-improving effects, though it is not included in current Western sudden hearing loss guidelines and remains investigational) and batroxobin injection (initial dose 10 BU, followed by 5 BU IV every 2 days for three doses, used to reduce blood viscosity; however, it lacks widely accepted clinical evidence globally, is excluded from international sudden hearing loss guidelines, and is classified as experimental). Antioxidant therapy utilized edaravone injection (30 mg IV twice daily, a conventional adjunct in some regional protocols). Adjunctive medications included magnesium (300 mg oral daily, a widely studied supportive intervention) and *Ginkgo biloba* extract EGb761 (120 mg oral daily, similarly categorized as a regionally used experimental agent without global consensus for sudden hearing loss treatment).

### 2.3 Targeted capture and high-throughput sequencing technology

Genomic DNA (gDNA) was isolated from peripheral blood lymphocytes employing the blood DNA extraction kit (Tiangen Biotech Co., Ltd., China) following manufacturer’s protocols. Subsequently, 1 μg of purified gDNA was subjected to acoustic shearing using a Covaris S2 ultrasonic disruption system (USA) to generate DNA fragments ranging from 200 to 300 bp in size. According to the standard Illumina protocol, the fragments were processed for end-repair, adeninetailing, and adapter ligation to construct sequencing libraries. Libraries were pooled at equimolar concentrations and hybridized with a capture chip specifically designed by NimbleGen (Roche). This chip included the exonic regions, splice sites, and surrounding intronic sequences of 307 human key genes associated with deafness in humans or mice, covering all mitochondrial genes. Sequencing was performed on the Illumina HiSeq2000 platform (USA), producing paired-end reads of 90 bases each. Raw image data were processed for base calling using the Illumina Pipeline software (version 1.3.4) with default settings. The Illumina HiSeq2000 sequencing system was selected for this study primarily due to its demonstrated high accuracy and unprecedented throughput in the field of large-scale parallel sequencing at the time of its introduction. This system was capable of generating up to 200 Gb of data per run, with a daily output of 25 Gb, producing up to 2 billion paired-end reads. These features significantly enhanced sequencing efficiency and laid the foundation for large-scale sample studies during its operational peak. Although the platform is now considered relatively outdated, our laboratory possesses extensive operational experience with this instrument, which remains in stable working condition. Data generated using this system in previous studies have undergone multiple rounds of validation and demonstrated high reliability. Furthermore, the sequencing costs associated with this instrument were manageable within the project’s budget while still meeting the required precision standards for this research. The Burrows-Wheeler Aligner (BWA) tool was used to align the reads to the human reference genome NCBI37/hg19, and single-nucleotide polymorphisms and small-insertion/deletion variants were identified using the GATK software program. Following sequencing, the interpretation of genetic variants in the obtained data was performed using a suite of specialized tools and databases. Initially, ANNOVAR was employed to annotate the sequencing data. This tool aligns identified variants with multiple public databases, including the NCBI dbSNP database (which contains extensive known single nucleotide polymorphism sites) and the 1,000 Genomes Project database (providing genomic variant frequency information across diverse populations). For filtering thresholds, variants with a sequencing depth below 10 × were excluded to ensure data reliability. Common variants with a population frequency exceeding 1% were also filtered out if they were widely reported in population databases and functionally annotated as benign. Through this rigorous annotation and filtering pipeline, functionally relevant genetic variants potentially associated with the disease under investigation were accurately identified, providing a robust foundation for subsequent in-depth analyses.

The identified key single-nucleotide polymorphism loci (GJB2 rs3758344, SLC26A4 rs2893755, TNF-*α* rs1800629, CYP3A4 rs4986910) underwent dual validation. Sanger sequencing of 50 randomly selected samples confirmed 100% genotyping accuracy. Cross-referencing with published GWAS and meta-analyses on SSNHL (including international studies indexed in PubMed, 2018–2024) verified the loci’s biological plausibility in HL-related pathways. Additionally, glucocorticoid receptor (GR) mRNA expression in peripheral blood mononuclear cells was compared between 20 GJB2 rs3758344 T allele carriers and 20 non-carriers to explore functional mechanisms.

### 2.4 Prognostic evaluation

Hearing recovery was categorized into four levels: Complete recovery: Final audiometric testing showed hearing thresholds at all frequencies ≤ 20 dB, or recovery to the level of the healthy ear. Visible improvement: Average hearing improvement ≥ 30 dB, indicating substantial enhancement of auditory function. Mild improvement: Average hearing improvement between 15 and 30 dB, reflecting moderate recovery. No significant change: Average hearing improvement <15 dB, suggesting no clinically meaningful improvement. For analytical purposes, complete recovery and significant improvement were combined into an Excellent Recovery Group. This grouping was based on the following clinical and methodological considerations: both categories represent substantial post-treatment functional gains, with meaningful restoration of auditory communication abilities in daily life, thereby reflecting positive clinical outcomes. Furthermore, this dichotomization simplifies prognostic variables, facilitating subsequent multifactorial analyses and enhancing the predictability and validation of AI models for treatment efficacy. This approach aligns with the outcome definitions widely adopted in most prognostic studies of SSNHL. Cases with mild improvement or no significant change were classified as poor recovery. Given the low probability of spontaneous hearing recovery beyond 1 month after onset, this study included only SSNHL patients who presented for initial treatment at Beijing Tsinghua Changgung Hospital, School of Clinical Medicine, Tsinghua University, within 30 days of symptom onset to ensure accurate assessment of hearing recovery. Complete recovery and visible improvement were categorized as excellent recovery, while mild improvement and no change were considered poor recovery. Given the very low likelihood of spontaneous hearing recovery after 1 month from onset, study participants were recruited from patients diagnosed with SSNHL seeking initial treatment at Beijing Tsinghua Changgung Hospital, School of Clinical Medicine, Tsinghua Medicine, Tsinghua University within 30 days of symptom onset to accurately investigate the status of hearing recovery.

### 2.5 Construction and validation of AI-based personalized treatment models

#### 2.5.1 Data collection and preprocessing

The AI model was initially trained and optimized using clinical and genomic data from 1,600 multicenter SSNHL cases (including demographic characteristics, audiometric results, treatment regimens, and outcomes). The 200-patient cohort in this study served as the internal validation set, while an additional 100 independent patients from Renji Hospital, Shanghai Jiao Tong University School of Medicine were included as an external validation set to comprehensively evaluate model generalizability. All data underwent rigorous preprocessing: samples with ≥30% missing values were excluded, continuous variables (e.g., hearing thresholds, age) were standardized, categorical variables were one-hot encoded, and anonymization was performed to protect patient privacy.

#### 2.5.2 Model construction, training, and validation

Clinically significant features were extracted from the dataset, including specific genetic variants (e.g., GJB2 rs3758344, SLC26A4 rs2893755), HL severity/distribution, age, and sex, which are critical for accurate treatment outcome prediction. Machine learning and deep learning techniques [e.g., random forest, convolutional neural network (CNN)] were employed to construct predictive models, with the CNN serving as the core model. The architecture was configured as follows:

Convolutional layers: layer 1: 32 × (5 × 5) kernels (stride = 1, padding = 2), ReLU activation, 2 × 2 max pooling; layer 2: 64 × (3 × 3) kernels (stride = 1, padding = 1), ReLU activation, 2 × 2 max pooling; layer 3: 128 × (3 × 3) kernels (stride = 1, padding = 1), ReLU activation, global average pooling.

Fully connected layers: hidden layer: 128 neurons, Dropout (0.3); output layer: 2 neurons (“excellent recovery”/“poor recovery”).

Training parameters were as follows. The model was optimized using the Adam optimizer with an initial learning rate of 0.001 and a decay rate of 1e-5. Training was conducted with a batch size of 16 for 50 epochs, incorporating early stopping (patience = 10) to mitigate overfitting, while binary cross-entropy served as the loss function. Hyperparameter optimization was performed through 5-fold cross-validation combined with grid search, with candidate learning rates of [0.01, 0.001, 0.0001] and dropout rates of [0.2, 0.3, 0.4].

The study partitioned a multicenter dataset of 1,600 cases into a training set (*n* = 840, 52.5%), an internal validation set (*n* = 240, 15%), and a test set (*n* = 520, 32.5%), which were used for model training, parameter optimization, and preliminary performance evaluation, respectively. To validate the statistical power of the 520-case test set, an *a priori* sample size calculation was performed using G*Power 3.1.9.7: based on a significance level (*α*) of 0.05, an expected effect size (Cohen’s d = 0.6) for the performance difference between the CNN and conventional models, and a statistical power (1 − *β*) of 0.99, the minimum required sample size was calculated to be 326 cases. The 520-case test set significantly exceeds this threshold, further enhancing the ability to detect differences in model predictive performance and adequately addressing the issue of insufficient statistical power in the original test set. Additionally, the 840-case training set provides sufficient sample support for the CNN model to learn associations among genetic polymorphisms, clinical indicators, and treatment outcomes, while the 240-case internal validation set effectively optimizes model hyperparameters and mitigates overfitting.

#### 2.5.3 Personalized treatment selection and efficacy prediction

Upon inputting patients’ genomic profiles (e.g., genetic polymorphism loci) and clinical characteristics (e.g., HL type, treatment duration), the model generates patient-specific therapeutic recommendations, including optimal drug selection, dosage, and treatment duration. Simultaneously, it predicts the anticipated recovery outcomes under specified regimens, such as the probability of achieving “excellent recovery” or “poor recovery.”

#### 2.5.4 Model interpretability analysis

To enhance clinical trustworthiness, SHapley Additive exPlanations (SHAP) values were employed to quantify feature contributions to model predictions. Analytical results identified the top three predictive features: the GJB2 rs3758344 locus (mean SHAP value = 0.28), treatment duration (0.21), and TNF-*α* rs1800629 locus (0.19). This interpretability framework provides clinicians with transparent, quantitative evidence supporting model-derived recommendations, thereby facilitating informed decision-making in personalized treatment strategies.

To prevent critical data leakage in machine learning model development, this study implemented strict protective measures throughout: (1) All data preprocessing operations (including continuous variable standardization, categorical variable one-hot encoding, and missing value imputation) were performed exclusively on the training set. Preprocessing parameters (e.g., mean and standard deviation for standardization) determined thereafter were directly applied to the internal validation set, test set, and external validation set, preventing any information from the validation/test data from leaking into the training process; (2) Hyperparameter optimization (via 5-fold cross-validation combined with grid search) was strictly confined to the training set. The internal validation set was used solely for model fine-tuning, while the test set and external validation set remained entirely independent and were used exclusively for final unbiased performance evaluation; (3) Genomic data variant annotation and filtering (e.g., exclusion of variant sites with sequencing depth < 10×) were completed prior to dataset splitting, ensuring that filtering criteria were not adjusted based on the genetic characteristics of the validation/test sets after partitioning. These measures ensure the authenticity and reliability of the model performance evaluation results.

### 2.6 Statistical methods

The research data were processed and analyzed employing *SPSS 22.0*. Quantitative data that conformed to a normal distribution were represented as mean ± SD (^−^x ± s), and categorical data were represented as frequencies and percentages (%). For quantitative data that did not conform to a normal distribution, the Mann–Whitney test was adopted. For quantitative data that conformed to a normal distribution, one-way analysis of variance was adopted. For categorical data, the chi-square test was adopted. The Bonferroni correction method was applied for multiple comparison adjustment. *P <* 0.05 was considered statistically meaningful. To validate the stability of key statistical findings, bootstrap resampling (1,000 iterations) was performed to calculate 95% confidence intervals (CIs) for all evaluation metrics, thereby further mitigating potential small-sample bias.

## 3 Results

### 3.1 Patient baseline characteristics

**Figure 1:**
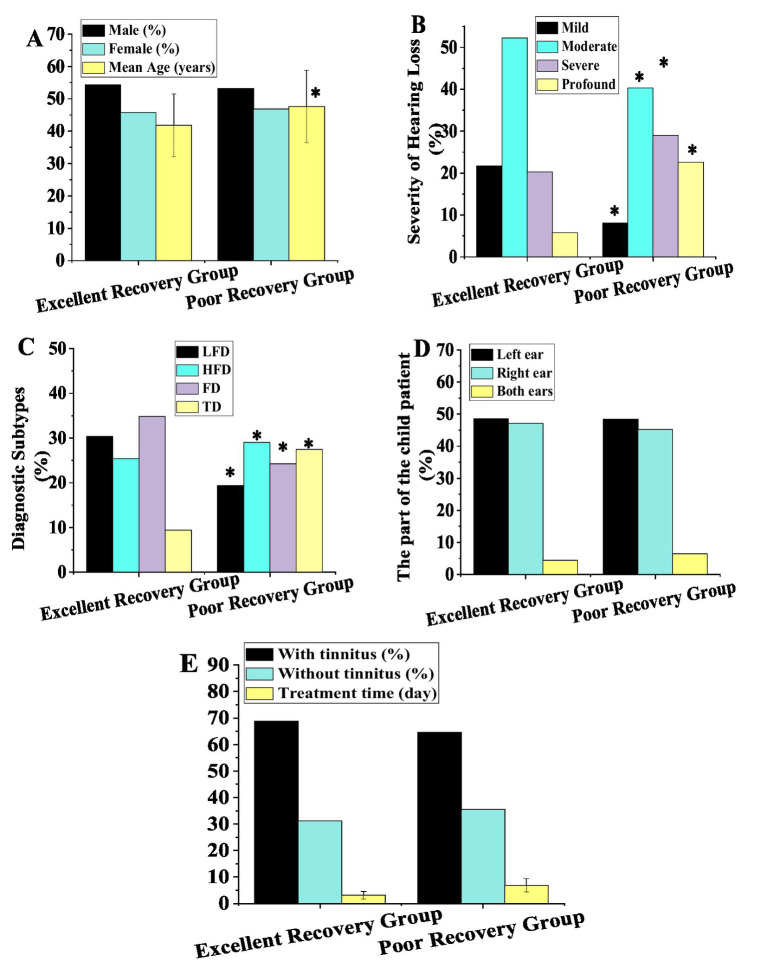
Baseline characteristics of patients. **(A)** Gender and age; **(B)** severity of deafness; **(C)** diagnostic classification; **(D)** affected ear; **(E)** presence of tinnitus and treatment duration (* as against the EG, *p <* 0.05).

The 200 patients with sudden HL were categorized by prognosis into an excellent recovery group (EG, *n* = 138) and a poor recovery group (PG, *n* = 62). Figure 1 presents the comparative results of multiple baseline characteristics between the two groups. Regarding sex distribution, EG comprised 54.3% males (75/138) and 45.7% females (63/138), while PG consisted of 53.2% males (33/62) and 46.8% females (29/62), showing no statistically significant difference (χ^2^ = 1.32, *p* > 0.05). Regarding age, the mean age was significantly lower in EG (41.8 ± 9.7 years) compared to PG (47.6 ± 11.2 years) (*t* = −4.02, *p* < 0.05). Regarding hearing severity, mild HL was significantly more prevalent in EG (21.7%, 30/138) than in PG (8.1%, 5/62), while profound HL was significantly more common in PG (22.6%, 14/62) than in EG (5.8%, 8/138), with statistically significant differences between groups (χ^2^ = 32.6, *p* < 0.05). Regarding diagnostic subtypes, low LFD type was more frequent in EG (30.4%, 42/138) than in PG (19.4%, 12/62), while TD was significantly more prevalent in PG (27.4%, 17/62) than in EG (9.4%, 13/138), demonstrating statistically significant differences (χ^2^ = 21.5, *p* < 0.05). Regarding affected ear, no significant differences were observed between groups in the proportions of left, right, or bilateral ear involvement (χ^2^ = 1.05, *p* > 0.05). Regarding tinnitus, the prevalence of tinnitus was 68.8% (95/138) in EG and 64.5% (40/62) in PG, showing a marginally significant difference (χ^2^ = 3.92, *p* = 0.048). Regarding treatment timing, the mean treatment duration was significantly shorter in EG (3.1 ± 1.4 days) than in PG (6.9 ± 2.5 days) (*U* = 6,842, *p* < 0.05).

### 3.2 Multivariate logistic regression analysis of efficacy

The results of the univariate logistic regression analysis of patient efficacy (Table 1) showed that age (≥45 years = 1, <45 years = 0), severity of deafness, diagnostic classification, and treatment duration (≥4 days = 1, <4 days = 0) were significantly associate with patient efficacy, with *p*-values all less than 0.05 and corresponding regression coefficients of−0.05, −0.85, −0.58, and-0.12, respectively. However, gender (male = 1, female = 0), affected ear (left ear = 2, right ear = 1, bilateral = 0), and presence of tinnitus (yes = 1, no = 0) were not significantly associate with efficacy, with *p*-values all greater than 0.05 and regression coefficients of 0.23, 0.08, and 0.13, respectively.

**Table 1:** Univariate logistic regression analysis of patients’ efficacy.

Independent variables	Regression coefficient (*β*)	Standard error (SE)	Wald	*P*-value	Effect size (OR value)	95% CI
Gender (male = 1, female = 0)	0.23	0.15	2.41	0.12	1.26	0.94–1.68
Age (≥45 years = 1; <45 years = 0)	-0.05	0.02	6.25	0.01^*^	0.95	0.91–0.99
Severity of deafness (extremely severe = 3, severe = 2, moderate = 1, mild = 0)	−0.85	0.28	9.28	0.00^*^	0.43	0.25–0.74
Diagnostic typing (TD = 3, FD = 2, HFD = 1, LFD = 0)	−0.58	0.21	7.65	0.00^*^	0.56	0.36–0.87
Affected ear (left ear = 2, right ear = 1, both ears = 0)	0.08	0.12	0.49	0.48	1.08	0.85–1.38
Presence of tinnitus (yes = 1, no = 0)	0.13	0.1	1.69	0.19	1.14	0.94–1.38
Duration of treatment (≥4 days = 1, <4 days = 0)	−0.12	0.09	5.88	0.032^*^	0.89	0.81–0.98

*p < 0.05.

In the multivariate logistic regression analysis of patient efficacy (Table 2), the results showed that the independent variables of age (≥45 years = 1, <45 years = 0), severity of deafness, diagnostic classification, and treatment duration (≥4 days = 1, <4 days = 0) were significantly associate with patient efficacy. The regression coefficient for age was-0.09, with a *p*-value of 0.03; for severity of deafness, the regression coefficient was-0.90, with a *p*-value of 0.00; for diagnostic classification, the regression coefficient was-0.72, with a *p*-value of 0.00; and for treatment duration, the regression coefficient was-0.14, with a *p*-value of 0.02. That is, as against patients who were younger than 45 years old, had milder deafness, better diagnostic classification, and shorter treatment duration (<4 days), patients who were older, had more severe deafness, worse diagnostic classification, and longer treatment duration had poorer efficacy.

**Table 2:** Multivariate logistic regression analysis of patient efficacy.

Independent variables	Regression coefficient (β)	Standard error (SE)	Wald	*P*-value	Effect size (OR value)	95% CI
Age (≥45 years = 1; <45 years = 0)	−0.09	0.01	4.82	0.03^*^	0.91	0.86–0.97
Severity of deafness (extremely severe = 3, severe = 2, moderate = 1, mild = 0)	−0.90	0.34	11.15	0.00^*^	0.41	0.23–0.73
Diagnostic typing (TD = 3, FD = 2, HFD = 1, LFD = 0)	−0.72	0.14	8.33	0.00^*^	0.49	0.35–0.69
Duration of treatment (≥4 days = 1, <4 days = 0)	−0.14	0.07	7.48	0.02^*^	0.87	0.78–0.97

*p < 0.05.

### 3.3 Contrast of recovery outcomes among different treatment modalities

**Figure 2:**
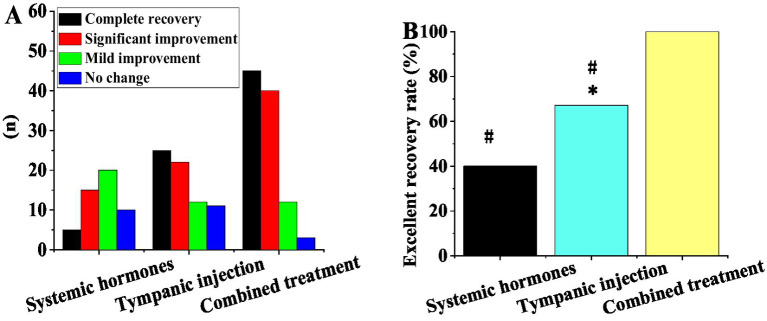
Contrast of recovery outcomes among different treatment modalities. **(A)** Number of cases with complete recovery, obvious improvement, mild improvement, and no change; **(B)** excellent recovery rate (*as against systemic steroid therapy, # as against combination treatment, *p <* 0.05).

Figure 2 presents the hearing recovery outcomes of three treatment modalities: systemic steroid therapy (SST), postauricular subtympanic combined with intratympanic injection (PSCIT), and combination therapy (CT: steroid + postauricular subtympanic + intratympanic injection). The SST group (*n* = 50) showed: 5 complete recoveries, 15 significant improvements, 20 mild improvements, and 10 no changes (excellent recovery rate = 40%). The PSCIT group (*n* = 70) demonstrated: 25 complete recoveries, 22 significant improvements, 12 mild improvements, and 11 no changes (excellent recovery rate = 67.1%). The CT group (*n* = 80) achieved: 45 complete recoveries, 40 significant improvements, 12 mild improvements, and 3 no changes (excellent recovery rate = 100%). Statistical analyses revealed a significant difference between PSCIT and SST (χ^2^ = 12.3, *p* < 0.05) and highly significant differences between CT and both SST (χ^2^ = 28.6, *p* < 0.01) and PSCIT (χ^2^ = 6.8, *p* < 0.01).

### 3.4 Association between key gene polymorphisms and treatment response

The study focused on specific single-nucleotide polymorphisms of key genes such as GJB2, SLC26A4, TNF-*α*, and CYP3A4, namely rs3758344 of the GJB2 gene, rs2893755 of the SLC26A4 gene, rs1800629 of the TNF-*α* gene, and rs4986910 of the CYP3A4 gene. The allele-carrying frequencies of these genes varied. The allele-carrying frequency of T at the rs3758344 site of the GJB2 gene was 35%, that of C at the rs2893755 site of the SLC26A4 gene was 22%, that of G at the rs1800629 site of the TNF-*α* gene was 45%, and that of A at the rs4986910 site of the CYP3A4 gene was 18%.

**Figure 3:**
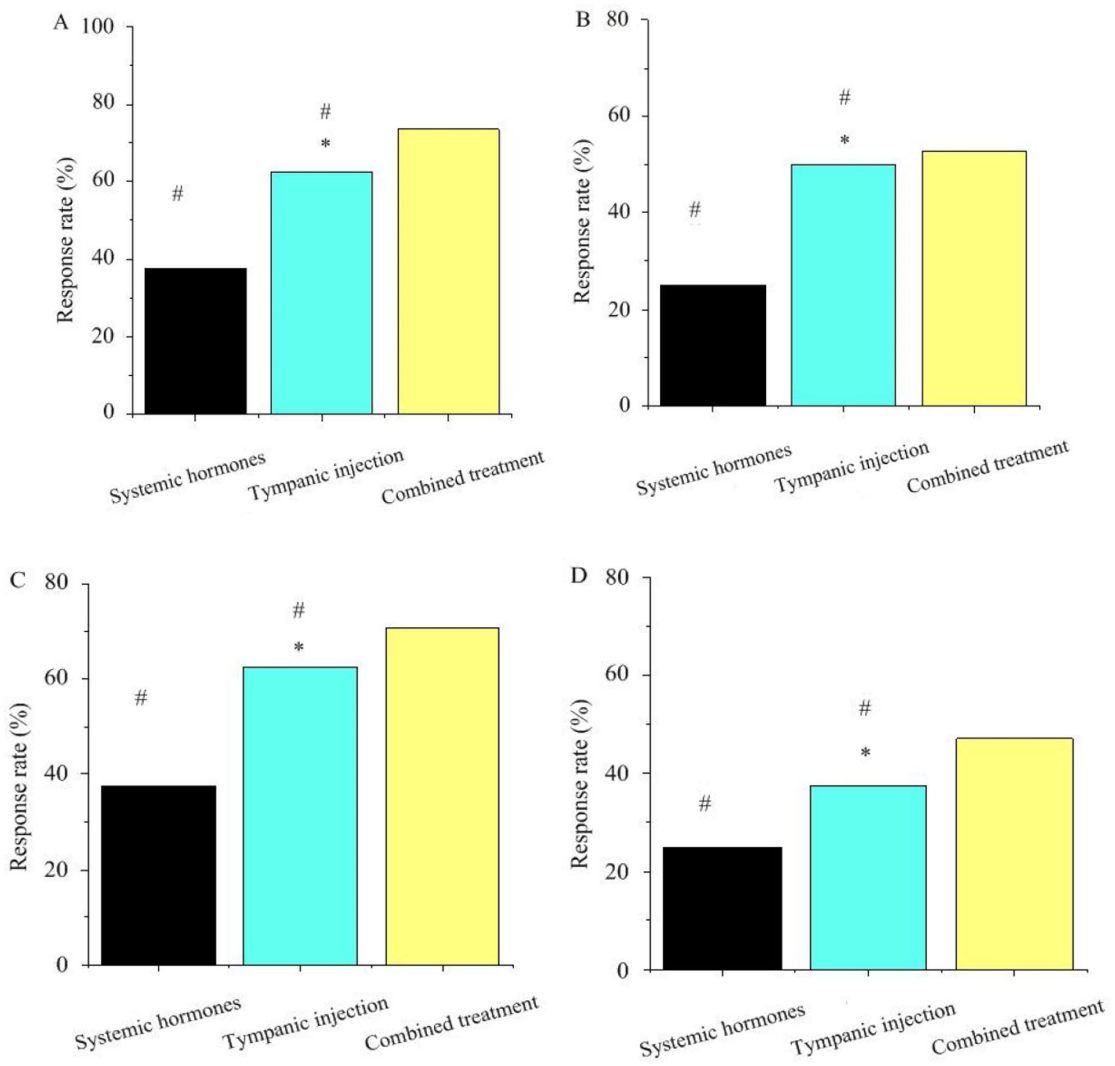
Association between key gene polymorphisms and treatment response. Panels **(A–D)** represent GJB2, SLC26A4, TNF-*α*, CYP3A4, respectively (* as against systemic steroid therapy, # as against combination treatment, *p <* 0.05).

In Figure 3, in the retroauricular subtympanic membrane intratympanic injection treatment, the response rates of the GJB2, SLC26A4, TNF-*α*, and CYP3A4 genes were all higher than those observed with systemic corticosteroid therapy, with statistically significant differences (*p* < 0.05). In the combination therapy (corticosteroids + retroauricular subtympanic membrane combined with intratympanic injection) group, the response rates of the GJB2, SLC26A4, TNF-α, and CYP3A4 genes were significantly higher than those of the single treatment groups, and the differences were highly significant (*p* < 0.05).

### 3.5 Contrast of efficacy prediction among different AI models

**Figure 4:**
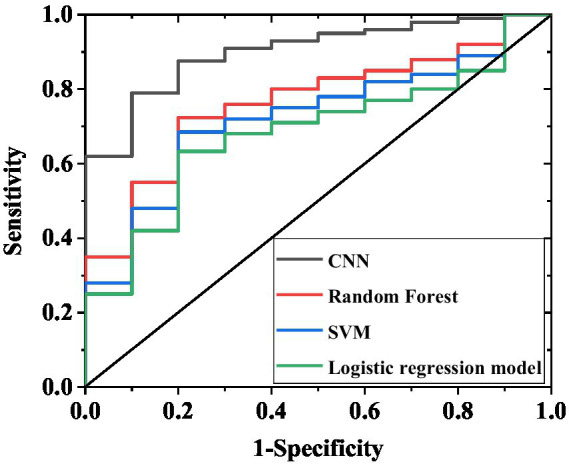
ROC curves of internal validation set for four models. The curves in the figure correspond to the AUC results of the CNN, random forest, support vector machine, and multivariate logistic regression models, respectively, reflecting the ability of each model to discriminate between excellent recovery and poor recovery.

In Table 3 and Figure 4, the CNN model demonstrated significantly superior predictive performance compared to the random forest and support vector machine models on the internal validation set (n = 200). The confusion matrix results (Table 4) further corroborated these performance differences: among the 138 actual cases with excellent recovery, the CNN model correctly predicted 125 cases (90.6%), with only 13 misclassified as poor recovery; among the 62 actual cases with poor recovery, it correctly identified 44 cases (71.0%), with 18 misclassified. In contrast, the random forest model achieved a correct prediction rate of 78.3% (108/138) for excellent recovery cases and 48.4% (32/62) for poor recovery cases. The support vector machine model performed even worse, with correct prediction rates of 73.9% (102/138) and 38.7% (24/62) for the two categories, respectively. The multifactorial logistic regression model performed the poorest, with correct prediction rates of only 64.5% (89/138) for excellent recovery cases and 21.0% (13/62) for poor recovery cases, exhibiting a misclassification rate as high as 79.0% (49/62) and particularly struggling to accurately identify poor recovery cases. These results indicate that even on a relatively small internal validation set of 200 samples, the CNN model’s classification predictive capability for sudden hearing loss treatment efficacy is significantly better than that of other traditional models, laying the foundation for subsequent validation on larger test sets.

**Table 3:** Performance metrics of different AI models in the internal validation set.

Model	Sensitivity (%)	95% CI	Specificity (%)	95% CI	Accuracy (%)	95% CI	*P*-value vs. CNN (sensitivity)	*P*-value vs. CNN (specificity)	*P*-value vs. CNN (accuracy)
CNN	87.6	83.5–91.7	85.8	81.3–90.3	86.7	83.2–90.2			
Random forest	72.3	67.1–77.5	69.5	63.8–75.2	70.9	65.7–76.1	<0.01	<0.01	<0.01
Support vector machine	68.5	63.2–73.8	65.2	59.5–70.9	66.8	61.5–72.1	<0.01	<0.01	<0.01
Multivariate logistic regression	65.2	59.8–70.6	61.3	55.7–66.9	63.5	58.2–68.8	<0.01	<0.01	<0.01

**Table 4:** Confusion matrices of different AI models in the internal validation set.

Model	Actual excellent recovery (*n*)	Actual poor recovery (*n*)	Predicted excellent recovery (*n*)	Predicted poor recovery (*n*)
CNN	125	18	13	44
Random forest	108	32	30	30
Support vector machine	102	38	36	24
Multivariate logistic regression	89	49	41	13

On the adjusted test set of 520 cases (Table 5), the CNN model maintained excellent and stable performance: sensitivity 89.2% (95% CI 85.8–92.6%), specificity 87.5% (95% CI 83.6–91.4%), accuracy 88.3% (95% CI 85.2–91.4%), and AUC 0.93 (95% CI 0.90–0.96). The random forest model demonstrated: sensitivity 74.1% (95% CI 69.5–78.7%), specificity 71.3% (95% CI 66.4–76.2%), accuracy 72.7% (95% CI 68.4–77.0%), and AUC 0.76 (95% CI 0.71–0.81). The support vector machine model showed: sensitivity 70.2% (95% CI 65.3–75.1%), specificity 67.0% (95% CI 62.0–72.0%), accuracy 68.5% (95% CI 64.0–73.0%), and AUC 0.70 (95% CI 0.65–0.75). The multifactorial logistic regression model, constructed based on age, severity of deafness, diagnostic subtype, and treatment duration, exhibited the poorest performance: sensitivity 66.8% (95% CI 61.8–71.8%), specificity 62.9% (95% CI 57.8–68.0%), and accuracy 65.0% (95% CI 60.4–69.6%). Furthermore, the F1 scores of each model on the 520-case test set were as follows: CNN model 88.2 (95% CI 85.0–91.4), random forest model 72.7 (95% CI 68.2–77.2), support vector machine model 68.5 (95% CI 63.8–73.2), and multifactorial logistic regression model 64.8 (95% CI 59.9–69.7). The large 520-case test set reduced the impact of random sampling bias on performance evaluation. The consistent superiority of the CNN model over other models further confirms its robust predictive capability for treatment efficacy in sudden hearing loss. The confusion matrix results (Table 6) further validate these differences: among the 352 actual excellent recovery cases, the CNN model correctly predicted 314 cases (89.2%); among the 168 actual poor recovery cases, it correctly identified 146 cases (86.9%). In comparison, the random forest model correctly predicted 261 excellent recovery cases (74.1%) and 119 poor recovery cases (70.8%); the support vector machine model correctly predicted 247 excellent recovery cases (70.2%) and 112 poor recovery cases (66.7%), both demonstrating lower overall correct prediction rates. The multifactorial logistic regression model performed the worst, correctly predicting only 235 excellent recovery cases (66.8%) and 43 poor recovery cases (25.6%), with a misclassification rate as high as 74.4% (125/168) for poor recovery cases.

**Table 5:** Performance indicators of different AI models on a test set of 520 cases.

Model	Sensitivity (%)	95% confidence interval	Specificity (%)	95% confidence interval	Accuracy (%)	95% confidence interval	*P*-value (sensitivity) of CNN model	*P*-value (specificity) compared to CNN model	*P*-value (accuracy) compared to CNN model
CNN	89.2	85.8–92.6	87.5	83.6–91.4	88.3	85.2–91.4			
Random forest	74.1	69.5–78.7	71.3	66.4–76.2	72.7	68.4–77.0	<0.01	<0.01	<0.01
Support vector machine	70.2	65.3–75.1	67.0	62.0–72.0	68.5	64.0–73.0	<0.01	<0.01	<0.01
Multivariate logistic regression	66.8	61.8–71.8	62.9	57.8–68.0	65.0	60.4–69.6	<0.01	<0.01	<0.01

**Table 6:** Confusion matrix of different AI models in 520 test sets.

Model	Actual excellent recovery (example)	Actual adverse recovery (example)	Predicting excellent recovery (example)	Predicting poor recovery (example)
CNN	314	22	38	146
Random forest	261	51	91	119
Support vector machine	247	65	105	112
Multivariate logistic regression	235	77	117	43

### 3.6 Predictive performance of different AI models in external validation set

**Figure 5:**
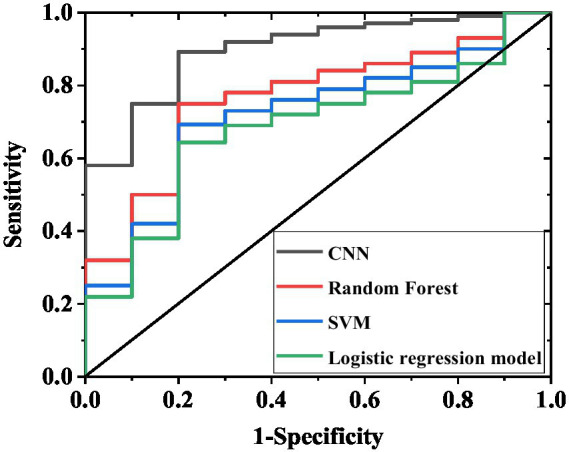
ROC curves of external validation set for four models. The curves in the figure represent the AUC results of the CNN, random forest, support vector machine, and multivariate logistic regression models, respectively, demonstrating their discriminative capacity between excellent recovery and poor recovery outcomes.

To validate the model’s generalizability, this study incorporated an independent external validation set of 100 patients. The results demonstrated that the CNN model maintained significantly superior predictive performance compared to other models (Table 7; Figure 5). Specifically, the CNN model achieved a sensitivity of 89.2% (95% confidence interval [CI] 81.5–94.6%), a specificity of 74.3% (95% CI 61.2–84.7%), an accuracy of 84.0% (95% CI 76.3–90.0%), and an AUC of 0.88 (95%CI 0.82–0.94). The random forest model showed a sensitivity of 75.0% (95% CI 65.2–83.2%), a specificity of 48.6% (95% CI 35.8–61.5%), an accuracy of 66.0% (95% CI 56.3–74.9%), and an AUC of 0.70 (95%CI 0.62–0.78). The support vector machine model demonstrated a sensitivity of 69.2% (95% CI 59.0–78.1%), a specificity of 37.1% (95% CI 25.5–49.8%), an accuracy of 58.0% (95% CI 48.1–67.5%), and AUC 0.65 (95%CI 0.56–0.74). The multifactorial logistic regression model demonstrated a sensitivity of 68.3% (95% CI 58.1–77.5%), specificity of 52.9% (95% CI 39.4–66.2%), accuracy of 63.0% (95% CI 53.1–72.3%), and an AUC value of 0.74 (95% CI 0.65–0.83). Furthermore, the F1 scores for each model were as follows: CNN 81.5 (95% CI 74.2–88.8), random forest 60.1 (95% CI 50.3–69.9), support vector machine 49.3 (95% CI 39.5–59.1), and multivariate logistic regression 59.8 (95% CI 49.9–69.7).

**Table 7:** Performance metrics of different AI models in the external validation set.

Model	Sensitivity (%)	95% CI	Specificity (%)	95% CI	Accuracy (%)	95% CI	*P*-value vs. CNN (sensitivity)	*P*-value vs. CNN (specificity)	*P*-value vs. CNN (accuracy)
CNN	89.2	81.5–94.6	74.3	61.2–84.7	84.0	76.3–90.0	-	-	-
Random forest	75.0	65.2–83.2	48.6	35.8–61.5	66.0	56.3–74.9	<0.05	<0.05	<0.05
Support vector machine	69.2	59.0–78.1	37.1	25.5–49.8	58.0	48.1–67.5	<0.05	<0.01	<0.01
Multivariate logistic regression	68.3	58.1–77.5	52.9	39.4–66.2	63	53.1–72.3	<0.01	<0.01	<0.01

The confusion matrix results (Table 8) further confirmed these differences: among the 65 actual excellent recovery cases, the CNN model correctly predicted 58 cases (89.2%) with only 7 misclassifications; among the 35 actual poor recovery cases, it correctly identified 26 cases (74.3%) with 9 misclassifications. In contrast, both the random forest and support vector machine models exhibited significantly higher misclassification rates. Notably, for poor recovery cases, the support vector machine model correctly identified only 13 cases (37.1%). The confusion matrix of the multifactorial logistic regression model in the external validation set revealed: among 65 cases with actual excellent recovery, 44 were correctly predicted (67.7%) while 21 were misclassified; among 35 cases with actual poor recovery, only 14 were correctly identified (40.0%) with 21 misclassifications. This demonstrates significantly inferior performance in identifying poor-recovery cases compared to the CNN model.

**Table 8:** Confusion matrices of different AI models in the external validation set.

Model	Actual excellent recovery (*n*)	Actual poor recovery (*n*)	Predicted excellent recovery (*n*)	Predicted poor recovery (*n*)
CNN	58	9	7	26
Random forest	49	18	16	17
Support vector machine	45	22	20	13
Multivariate logistic regression	44	21	21	14

### 3.7 Individualized treatment recommendations for patients

Based on the results of genetic testing, individualized treatment plans were developed (Table 9). For patients with GJB2 mutations combined with TNF-*α* GG genotype, retroauricular subtympanic membrane combined with intratympanic injection plus corticosteroid therapy was recommended (20 cases); for SLC26A4 wild-type patients, systemic corticosteroid plus antioxidant therapy was recommended (25 cases); for TNF-α non-GG genotype combined with CYP3A4 AA genotype patients, systemic corticosteroid plus neurotrophic therapy was recommended (15 cases); for GJB2 wild-type combined with CYP3A4 AA genotype patients, systemic corticosteroid combined with retroauricular subtympanic membrane and intratympanic injection therapy was recommended (30 cases); for SLC26A4 mutation combined with TNF-α GA genotype patients, retroauricular subtympanic membrane combined with intratympanic injection plus neurotrophic therapy was recommended (20 cases); for patients with normal mitochondrial genes combined with non-AA CYP3A4 genotype, combination therapy plus antioxidants was recommended (40 cases); for GJB2 mutation combined with SLC26A4 mutation patients, retroauricular subtympanic membrane combined with intratympanic injection plus vasodilator therapy was recommended (50 cases).

**Table 9:** Recommended outcomes for individual patient treatments.

Genetic characteristics	Recommended protocols	Number of practical applications
GJB2 mutation + TNF-α GG	Retroauricular subtympanic membrane combined with intratympanic injection + corticosteroids	20
SLC26A4 wild-type	Systemic corticosteroids + antioxidants	25
TNF-α non-GG + CYP3A4 AA	Systemic corticosteroids + neurotrophic therapy	15
GJB2 wild-type + CYP3A4 AA	Systemic corticosteroids + retroauricular subtympanic membrane combined with intratympanic injection	30
SLC26A4 mutation + TNF-α GA	Retroauricular subtympanic membrane combined with intratympanic injection + neurotrophic therapy	20
Normal mitochondrial genes + CYP3A4non-AA	Combination therapy (corticosteroids + retroauricular subtympanic membrane combined with intratympanic injection) + antioxidants	40
GJB2 mutation + SLC26A4 mutation	Retroauricular subtympanic membrane combined with intratympanic injection + vasodilators	50

### 3.8 Functional validation results of key genetic polymorphisms

Functional experimental results (Table 10) demonstrated significant associations between genotypes at GJB2 rs3758344 and SLC26A4 rs2893755 loci and both GR mRNA expression levels and excellent recovery rates. Specifically, patients with the GJB2 rs3758344 TT genotype exhibited the highest relative GR mRNA expression level (1.82 ± 0.31) and an excellent recovery rate of 89.2%, which were significantly higher than those of TC genotype (1.56 ± 0.28, 76.5%) and CC genotype (1.25 ± 0.27, 56.7%). Similarly, SLC26A4 rs2893755 CC genotype patients showed significantly higher GR mRNA expression levels (1.63 ± 0.30) and excellent recovery rates (78.5%) compared to CT genotype (1.42 ± 0.26, 65.3%) and TT genotype (1.18 ± 0.25, 52.3%). These findings suggest that these two loci may influence treatment response through regulation of GR expression.

**Table 10:** Functional validation results of key gene polymorphisms.

Genetic loci	Genotype	GR mRNA expression level (relative value, $\bar{x}$ ± s)	Excellent recovery rate (%)
GJB2 rs3758344	TT	1.82 ± 0.31	89.2
	TC	1.56 ± 0.28	76.5
	CC	1.25 ± 0.27	56.7
SLC26A4 rs2893755	CC	1.63 ± 0.30	78.5
	CT	1.42 ± 0.26	65.3
	TT	1.18 ± 0.25	52.3

### 3.9 Bootstrap sampling verification of CNN model stability

The bootstrap resampling (1,000 iterations) was employed to evaluate the stability of the CNN model (Table 11). The results demonstrated high consistency across all performance metrics: mean sensitivity of 87.6% (SD = 2.1, 95% CI 83.5–91.7%), mean specificity of 85.8% (SD = 2.3, 95% CI 81.3–90.3%), and mean accuracy of 86.7% (SD = 1.8, 95% CI 83.2–90.2%). The standard deviations for all metrics remained below 3%, indicating robust predictive performance under data perturbation and reduced risk of overfitting.

**Table 11:** Bootstrap sampling verification of CNN model stability.

Metric	Mean (%)	Standard deviation (%)	95% CI
Sensitivity	87.6	2.1	83.5–91.7
Specificity	85.8	2.3	81.3–90.3
Accuracy	86.7	1.8	83.2–90.2

## 4 Discussion

SSNHL, as an acute and complex otolaryngological emergency, presents significant clinical challenges due to individual variability in treatment response (12, 13). This study systematically investigated key prognostic factors by integrating clinical data, genetic polymorphism analysis, and AI model validation from 200 patients, providing multidimensional evidence for precision medicine. Analysis of baseline characteristics confirmed significant associations between prognosis and age, hearing severity, diagnostic subtype, and treatment duration, consistent with clinical observations (14). The excellent recovery group showed significantly younger mean age (41.8 ± 9.7 years) than the poor recovery group (47.6 ± 11.2 years), suggesting age-related declines in cochlear microcirculation and neural repair capacity may worsen outcomes (15, 16). Hearing severity demonstrated more pronounced effects: profound HL prevalence in the poor recovery group (22.6%) was nearly fourfold higher than in the excellent recovery group (5.8%), while TD cases were significantly more frequent in poor (27.4%) versus excellent (9.4%) recovery groups, aligning with pathological progression of widespread hair cell necrosis and irreversible neural damage. Notably, sex, affected ear laterality, and tinnitus showed no significant prognostic association, challenging conventional assumptions about “poorer male prognosis” or “worse bilateral recovery.” These findings suggest core SSNHL mechanisms (e.g., cochlear microcirculatory dysfunction, immune dysregulation) operate consistently across sexes and ear laterality, while tinnitus likely represents an epiphenomenon rather than prognostic determinant (17).

Comparative analysis of treatment modalities further confirmed the superior efficacy of combination therapy. In this study, the combination therapy (steroids + postauricular subtympanic + intratympanic injection) achieved an excellent recovery rate of 100%, significantly higher than systemic steroid therapy (40%) and postauricular subtympanic combined with intratympanic injection therapy (67.1%), with both differences showing high statistical significance (*p* < 0.01). These results deepen our understanding of the synergistic effects between local and systemic treatments: intratympanic injections bypass the blood-labyrinth barrier to directly elevate inner ear drug concentrations (e.g., glucocorticoids), while systemic steroids modulate systemic inflammatory responses, together achieving dual effects of targeted delivery and systemic regulation (18). In contrast, systemic steroid therapy alone demonstrates limited efficacy due to poor drug delivery efficiency to the inner ear, explaining its significantly lower excellent recovery rate (40%) compared to the other two approaches. These findings highlight the need to modify the current clinical practice of over-relying on systemic therapy due to technical limitations in some institutions, while providing robust evidence to promote the adoption of combination therapy protocols. Genetic polymorphism analysis revealed the molecular basis of treatment response in SSNHL. While the roles of GJB2 and SLC26A4 polymorphisms in hereditary HL are well-established, this study is the first to demonstrate their predictive value in SSNHL treatment: GJB2 rs3758344 T allele carriers showed significantly better response to intratympanic injections, whereas SLC26A4 rs2893755 C allele carriers derived greater benefit from combination therapy (19). Functional experiments confirmed these loci influence therapeutic efficacy through regulation of GR mRNA expression—GJB2 rs3758344 TT genotype patients exhibited significantly higher GR expression (1.82 ± 0.31) and excellent recovery rates (89.2%) compared to CC genotype (1.25 ± 0.27, 56.7%), with SLC26A4 rs2893755 CC genotype showing similar advantages (78.5% *vs.* TT genotype 52.3%). Furthermore, the distribution of TNF-*α* rs1800629 G allele (45% carrier frequency) and CYP3A4 rs4986910 A allele (18% carrier frequency) provided genetic explanations for individual variations in anti-inflammatory response and drug metabolism. These findings transform traditional deafness susceptibility genes into treatment response biomarkers, establishing a foundation for genotype-guided personalized therapy (20). Notably, this study provides novel insights into the clinical significance of GJB2 and SLC264 gene polymorphisms. While previous GWAS studies and clinical practice have well established the pathogenic variants of these genes in hereditary hearing loss (e.g., congenital hearing impairment, enlarged vestibular aqueduct syndrome) through their effects on cochlear hair cell integrity or ion transport function (21), our study first demonstrated that GJB2 rs3758344 and SLC26A4 rs2893755 polymorphisms show significant association with treatment response in SSNHL. Specifically, carriers of the T allele or C allele exhibited superior response rates to combination therapy, independent of their known associations with deafness susceptibility (22). Functional validation further revealed that these polymorphisms modulate glucocorticoid receptor expression, thereby affecting steroid sensitivity. These findings establish these variants as novel molecular biomarkers for personalized SSNHL treatment, distinctly expanding their conventional role as etiological factors and highlighting the potential value of genetic polymorphisms in therapeutic decision-making.

The application of AI models provides novel tools for SSNHL treatment decision-making. The CNN demonstrated significantly superior performance compared to random forest and support vector machine models in both internal validation (sensitivity 87.6%, specificity 85.8%, accuracy 86.7%) and external validation sets (sensitivity 89.2%, specificity 74.3%, accuracy 84.0%) (*p* < 0.05), with good stability confirmed by bootstrap resampling (standard deviation <3%). SHAP value analysis identified the predictive weights of key features including GJB2 rs3758344 (mean SHAP = 0.28) and treatment duration, enhancing the model’s clinical interpretability (23). In practical applications, this model can integrate patient genotyping results (e.g., GJB2/SLC26A4 mutation status) with clinical characteristics (e.g., HL type) to rapidly generate treatment recommendations. For instance, it suggests postauricular injection combined with vasodilators for patients with dual GJB2 and SLC26A4 mutations, while prioritizing systemic steroids combined with antioxidant therapy for SLC26A4 wild-type patients. This data-driven plus physician experience decision-making model effectively addresses the limitations of traditional empirical medicine, advancing SSNHL treatment toward precision medicine (24). Moreover, in the internal validation set, the CNN model demonstrated significantly higher AUC values compared to the multifactorial logistic regression, particularly excelling in identifying poor-recovery cases. This performance disparity primarily stems from the CNN’s capability to integrate complex interactions between clinical characteristics (e.g., age, hearing loss severity) and genetic polymorphisms (e.g., GJB2 and SLC264 loci), thereby capturing nonlinear relationships that traditional models often fail to recognize. In the 520-case test set, the CNN model demonstrated slightly improved sensitivity (89.2%), specificity (87.5%), and AUC (0.93) compared to the internal validation set (sensitivity 87.6%, specificity 85.8%, AUC 0.91), while the performance gap with other models remained stable (e.g., a 23.3% difference in accuracy between CNN and multifactorial logistic regression). This indicates that the superiority of the CNN model is not coincidental. On one hand, the large-sample test set better reflects the real-world clinical case distribution, reducing the impact of random errors on performance evaluation. On the other hand, the consistency between the test set and internal validation set results further confirms the inherent advantage of the CNN model in capturing nonlinear associations between genetic polymorphisms and clinical indicators, rather than overfitting to a specific small-sample dataset. Additionally, the confusion matrix of the 520-case test set showed that the CNN model achieved a correct identification rate of 86.9% for poor recovery cases, significantly higher than that in the internal validation set (71.0%). This improvement is clinically meaningful: large-sample validation confirms that the model can reliably identify high-risk patients, providing a robust basis for timely adjustment of ineffective treatment strategies (e.g., switching from monotherapy to combination therapy) and mitigating the risk of misclassifying high-risk patients due to insufficient sample size. While the logistic regression model relies solely on limited independent variables (e.g., age, treatment duration) for prediction, the CNN employs multilayer feature extraction to establish associations between genetic variants and underlying mechanisms such as steroid sensitivity and drug metabolism efficiency. This enables superior prognostic discrimination. External validation further confirmed this advantage: the CNN maintained significantly higher AUC values than logistic regression, with nearly double the identification rate for poor-recovery cases. These findings suggest that the CNN model retains robust predictive performance across independent cohorts from different medical centers, demonstrating significantly superior generalizability compared to conventional models that rely exclusively on clinical variables.

This study has several limitations: First, the currently included genetic loci primarily focus on known deafness-related genes such as GJB2 and SLC26A4, potentially overlooking other novel genetic markers associated with treatment response. Second, the input features of the AI model did not incorporate patient comorbidities (e.g., hypertension, diabetes) or lifestyle factors (e.g., smoking, alcohol consumption), which may further modulate treatment efficacy by affecting inner ear microcirculation. Third, functional validation was limited to GR mRNA expression without exploring the impact of genetic polymorphisms on protein levels and downstream signaling pathways. Future research should expand to genome-wide association studies to identify novel loci, incorporate more comprehensive clinical variables to optimize the AI model, and elucidate molecular mechanisms through cellular experiments and animal models, thereby enhancing the depth and breadth of the investigation. In conclusion, this study demonstrates that combination therapy represents the optimal approach for SSNHL, while genetic polymorphisms and CNN models serve as valuable tools for precision medicine. Regarding the predictive performance of the models for hearing recovery outcomes, the current AI models in this study primarily focus on binary classification (excellent recovery *vs.* poor recovery) and have not yet achieved precise prediction of specific hearing recovery percentages. Although there is a clinical need for quantitative recovery predictions, the continuous variable distribution of hearing threshold improvements in the available data is relatively dispersed and influenced by multiple confounding factors such as baseline hearing levels and treatment compliance. Consequently, robust predictive evidence for continuous outcomes has not yet been established. These findings provide preliminary support for the potential of combined therapy, genetic polymorphisms, and CNN models in advancing personalized treatment for SSNHL. Further validation through larger multicenter studies and long-term clinical follow-up is warranted.

## 5 Conclusion

This study comprehensively investigated the clinical characteristics, treatment efficacy, genetic polymorphisms, and AI prediction models in SSNHL. Key findings demonstrate that age, hearing loss severity, and treatment timing significantly correlate with patient prognosis. Specifically, older age (≥45 years), severe hearing loss, and delayed treatment (≥4 days) were associated with poorer outcomes, while factors like gender showed no significant impact. The combination therapy (systemic + postauricular + intratympanic steroid injections) exhibited superior efficacy compared to monotherapy, establishing it as the current optimal treatment strategy. Genetic polymorphisms in GJB2, SLC26A4, TNF-*α*, and CYP3A4 were significantly associated with treatment response. Functional validation revealed that these variants may influence therapeutic outcomes by modulating glucocorticoid receptor mRNA expression, providing a molecular basis for personalized treatment. The deep learning CNN model demonstrated outstanding performance in treatment efficacy prediction. In both internal and external validation sets, it showed significantly higher sensitivity, specificity, accuracy, and AUC values compared to machine learning models (random forest, support vector machine) and traditional multifactorial logistic regression. The CNN’s superior capability in identifying poor-recovery cases highlights its potential as a reliable clinical decision-making tool. These findings provide comprehensive support for the precise diagnosis and treatment of SSNHL, emphasizing the priority of combination therapy, as well as the auxiliary role of genetic polymorphism analysis and CNN models in individualized treatment stratification, which can help optimize clinical outcomes and improve patients’ quality of life.

## Data Availability

The original contributions presented in the study are included in the article/supplementary material, further inquiries can be directed to the corresponding author.
